# Financial Hardship Among Patients With Early-Stage Colorectal Cancer

**DOI:** 10.1001/jamanetworkopen.2024.31967

**Published:** 2024-09-17

**Authors:** Gelareh Sadigh, Fenghai Duan, Na An, Ilana D. Gareen, JoRean Sicks, Jennifer M. Suga, Heather Kehn, Paul T. Mehan, Rajesh Bajaj, David S. Hanson, Samir M. Dalia, Jared D. Acoba, Demet GoKalp Yasar, Michael A. Taylor, Elyse Park, Lynne I. Wagner, Sheetal M. Kircher, Ruth C. Carlos

**Affiliations:** 1 University of California Irvine School of Medicine; 2 Department of Biostatistics, Brown University School of Public Health, Providence, Rhode Island; 3 Center for Statistical Sciences, Brown University School of Public Health, Providence, Rhode Island; 4 Department of Epidemiology, Brown University School of Public Health, Providence, Rhode Island; 5 Kaiser Permanente NCI Community Oncology Research Program and NCORP, Vallejo, California; 6 Metro-Minnesota Community Oncology Research Consortium, St Louis Park; 7 Missouri Baptist Hospital NCORP, St Louis; 8 Carolina Health Care and NCORP, Florence, South Carolina; 9 Mary Bird Perkins Cancer Center, Baton Rouge, Louisiana; 10 Mercy Hospital, Joplin, Missouri; 11 University of Hawaii Cancer Center, Honolulu; 12 Marshfield Clinic, Minocqua, Wisconsin; 13 PeaceHealth St Joseph Medical Center, Bellingham, Washington; 14 Massachusetts General Hospital Cancer Center, Boston; 15 Wake Forest University Health Sciences, Winston-Salem, North Carolina; 16 Robert H. Lurie Comprehensive Cancer Center of Northwestern University, Chicago, Illinois; 17 University of Michigan Comprehensive Cancer Center, Ann Arbor

## Abstract

**Importance:**

The degree of cancer patients’ financial hardship is dynamic and can change over time.

**Objective:**

To assess longitudinal changes in financial hardship among patients with early-stage colorectal cancer.

**Design, Setting, and Participants:**

In this prospective longitudinal cohort study, English-speaking adult patients with a new diagnosis of stage I to III colorectal cancer being treated with curative intent at National Cancer Institute (NCI) Community Oncology Research Program (NCORP) practices between May 2018 and July 2020 and who had not started chemotherapy and/or radiation were included. Data analysis was conducted from March to December 2023.

**Main Outcomes and Measures:**

Patients completed surveys at baseline as well as at 3, 6, 12, and 24 months after enrollment. Cost-related care nonadherence and material hardship, as adopted by Medical Expenditure Panel Survey, were measured. Factors associated with financial hardship were assessed using longitudinal multivariable logistic regression models with time interaction.

**Results:**

A total of 451 patients completed baseline questions, with 217 (48.1%) completing the 24-month follow-up. Mean (SD) age was 61.0 (12.0) years (210 [46.6%] female; 33 [7.3%] Black, 380 [84.3%] White, and 33 [7.3%] American Indian or Alaska Native, Asian, multiracial, or Native Hawaiian or Other Pacific Islander individuals or those who did not report race or who had unknown race). Among 217 patients with data at baseline and 24 months, 19 (8.8%) reported cost-related care nonadherence at baseline vs 20 (9.2%) at 24 months (*P* = .84), and 125 (57.6%) reported material hardship at baseline vs 76 (35.0%) at 24 months (*P* < .001). In multivariable analysis, lower financial worry (odds ratio [OR], 0.90; 95% CI, 0.87-0.93), higher education (OR, 0.34; 95% CI, 0.15-0.77), and older age (OR, 0.94; 95% CI, 0.91-0.98) were associated with lower nonadherence. Receipt of chemotherapy was associated with higher material hardship (OR, 2.68; 95% CI, 1.15-6.29), while lower financial worry was associated with lower material hardship (OR, 0.83; 95% CI, 0.80-0.96). Over 24 months, female sex was associated with lower nonadherence (OR, 0.90; 95% CI, 0.85-0.96), while higher education was associated with higher nonadherence (OR, 1.09; 95% CI, 1.03-1.17). Being employed was associated with lower material hardship (OR, 0.85; 95% CI, 0.78-0.93), while receipt of care at safety-net hospitals was associated with higher hardship (OR, 1.09; 95% CI, 1.01-1.17).

**Conclusions and Relevance:**

In patients with early-stage colorectal cancer, material hardship was more common than cost-related cancer care nonadherence and decreased over time, while nonadherence remained unchanged. Early and longitudinal financial screening and referral to intervention are recommended to mitigate financial hardship.

## Key Points

**Question:**

Do rates of financial hardship change among patients with early-stage colorectal cancer over time?

**Findings:**

In this cohort study of 451 patients with a new diagnosis of stage I to III colorectal cancer treated with curative intent, overall cost-related care nonadherence did not significantly change over 24 months, while material hardship significantly decreased.

**Meaning:**

In patients with early-stage colorectal cancer, material hardship was more common than cost-related care nonadherence and decreased over time, while nonadherence remained unchanged.

## Teaser

This cohort study examines whether there are changes in cost-related care nonadherence and financial hardship among US patients with early-stage colorectal cancer over a 2-year period.

## Introduction

More than 50% of cancer survivors report financial hardship,^1^ characterized by 3 domains: cost-related care nonadherence, material hardship, and financial worry, which are linked to poor quality of life, increased symptom burden, and decreased survival.^2,3,4,5,6^ The degree of financial hardship among patients with cancer is dynamic, with changes in patients’ treatment, employment, insurance, and income because of their inability to work due to cancer or cancer treatment. Few longitudinal studies of financial hardship in cancer focus either on changes in financial worry or material hardship. Shankaran et al^7^ showed patients with newly diagnosed metastatic colorectal cancer experience a cumulative incidence of 71.3% material hardship at 12 months after enrollment. In a nationally representative database,^8^ 42.4% of patients with newly diagnosed cancer depleted their entire life assets at 2 years after diagnosis. Eighty-two percent of patients with breast cancer at Mayo Clinic Rochester reported stability or improvement in financial concerns within an average of 25.6 months after diagnosis; while 18% had worsening financial concerns.^9^ Furthermore, in patients with breast cancer, financial distress after breast cancer treatment significantly decreased compared with during treatment, but remained higher than before treatment initiation.^10^

We expect that patients with early-stage cancer experience financial hardship but at a lower rate than metastatic cancer due to difference in intensity and duration of treatment. Changes in financial hardship, specifically cost-related care nonadherence and material hardship, have not been studied before. We aimed to assess longitudinal changes in financial hardship among patients with early-stage colorectal cancer treated at National Cancer Institute (NCI) Community Oncology Research Program (NCORP) practices across the United States.

## Methods

EAQ162CD was a prospective, longitudinal observational cohort study coordinated by the ECOG-ACRIN Cancer Research Group. It was approved by the NCI Central institutional review board. EAQ162CD is registered at ClinicalTrials.gov (NCT03516942), and all patients provided written informed consent. Data from EAQ162CD are available by contacting ECOG-ACRIN. This article follows the Strengthening the Reporting of Observational Studies in Epidemiology (STROBE) reporting guideline.

### Study Population

Participants were enrolled through NCORP sites and were eligible if they were aged 18 years or older, were English-speaking, had a new diagnosis of colon or rectal cancer within 60 days of registration in the study and had not yet started chemotherapy or radiation, and were stage I to III and being treated with curative intent. Patients with Eastern Cooperative Oncology Group (ECOG) performance scores of 4 and without the capacity to consent were excluded.

### Design

Between May 2018 and July 2020, a convenience sample of eligible patients was approached to participate in the study, as previously described.^11^ Participants completed a 30-minute paper or online survey either in the clinic or at home and were followed up at 3, 6, 12 and 24 months after enrollment. Follow-up surveys were completed electronically or on paper through mail. Patients who did not return surveys were contacted by telephone. In total, 565 patients were registered between May 2018 and July 2020. The study flow diagram is shown in the Figure. Only patients who completed at least 1 of the questions on care nonadherence or material hardship at baseline were included (N = 451).

**Figure.**
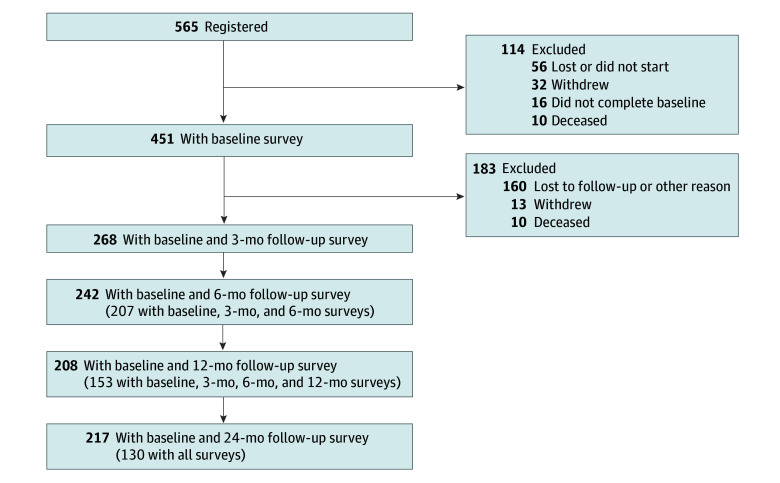
Study Flow Diagram

### Survey Measures

Our survey measures included instruments to assess financial worry using Comprehensive Score for Financial Toxicity (COST; score ranging from 0-44, with lower score indicating worse financial worry),^12^ cost-related care nonadherence and material financial hardship as adapted from Medical Expenditure Panel Survey (MEPS),^13,14,15^ self-efficacy,^16^ demographics, and health insurance. Cost-related care nonadherence was defined as at least 1 report of delay, foregoing, or change in adherence to medication or refusal of tests due to medical costs during the prior 3 months. If any of these were answered with yes, then care nonadherence was considered a yes.

Material financial hardship was defined as any reported incidence of minor hardship (decreased basic spending on food, clothing, or leisure activities) or major hardship (withdrawals from retirement or savings accounts, borrowing money to pay for cancer care, or patient or family member working more to pay for cancer care) during the prior 3 months. If any of these were answered with yes, then material hardship was considered a yes.

The change in material hardship was defined by comparing the number of major or minor conditions between baseline and the 24 months. If major conditions were reported, the change in their numbers define improvement (fewer major conditions at 24 months than at baseline), no change (an equal number of major conditions at both times), or worsening (more major conditions at 24 months than at baseline). If no major conditions were reported at either time, then the minor conditions define improvement, no change, or deterioration following the same criteria. Change in care nonadherence was defined as improvement (nonadherence at baseline, but no nonadherence at 24 months), no change, or worsening (no nonadherence at baseline, but nonadherence at 24 months).

Self-efficacy was measured using a 6-item Stanford Self-Efficacy for Management of Chronic Disease (score range 1-10, with higher score indicating better self-efficacy).^16^ Sociodemographic and clinical variables including self-reported age, sex, ethnicity, race, education, marital status, income, employment status, disability status, region, insurance type, cancer type or stage, and comorbidities were collected. Neighborhood area deprivation index (ADI) score was calculated using the Agency for Healthcare Research and Quality socioeconomic status index.^17,18^ The index (range, 0-100) represents a weighted combination of wealth, income, education, occupation, and housing conditions,^17^ with higher values corresponding to greater neighborhood-level socioeconomic disadvantage.^19,20^

### Statistical Analysis

Descriptive statistics were generated for all numeric variables using mean and SD or median and quartiles and for all categorical variables using frequencies and percentages. Model assumptions were assessed and verified. The longitudinal analysis of care nonadherence or material hardship in the generalized linear mixed model for binary outcomes was performed to include measurements at 5 visits (baseline, 3, 6, 12, and 24 months). The covariates included baseline variables (eg, age, sex, ethnicity, race, marital status, education, region, household income, primary insurance, employment, cancer type, cancer stage, comorbidities, ADI, COST, and safety-net hospital), time, and chemotherapy plan that was informed by baseline and 3-month data. These covariates represent sociodemographic and clinical variables that are hypothesized to moderate the outcomes, using the theoretical model of financial hardship developed by Yabroff et al.^21^ Race was self-reported as American Indian or Alaska Native, Asian, Black, multiracial, Native Hawaiian or Other Pacific Islander, White, not reported, and unknown. American Indian or Alaska Native, Asian, multiracial, and Native Hawaiian or Other Pacific Islander individuals were combined with those who did not report race or whose race was unknown into a separate group (other race) due to small sample sizes. Ethnicity was self-reported as Hispanic or Latino non-Hispanic or Latino, not reported, and unknown. Longitudinal models used an unstructured covariance structure by including a random intercept for each participant. The covariates were modeled as fixed effects. The longitudinal model of trajectory used continuous time (in months) grouped by the 5 time points. Two-way interactions between each covariate and the time were assessed prior to the main-effect modeling and the significant interactions were kept in the final models.

A sensitivity analysis was conducted to use inverse probability weighting (IPW) to assess the impact of missing values on the 2 longitudinal models. The method involves weighting individuals by the inverse of their probability of having nonmissing data, based on the set of observed covariates. This helps to mitigate the biases associated with nonrandom missingness, facilitating more robust and valid inferences from the analyzed data.^22^ Statistical significance was *P* < .05, and statistical analyses were performed using R version 4.4.0 (R Project for Statistical Computing) and SAS version 9.4 (SAS Institute Inc). For categorical covariates, the significance was based on the overall 2-sided *P* value (or type III *P* value). Therefore, no correction for multiple comparisons was applied during the analyses.

## Results

A total of 565 patients from 172 practices were enrolled in the longitudinal study, of these 451 (79.8%) completing at least 1 of the 9 survey questions on cost-related care nonadherence and material hardship at baseline (Figure), and a total of 268 (47.4%), 242 (42.8%), 208 (36.8%), and 217 (38.4%) completed those surveys at 3, 6, 12 and 24 months. Survey completion was similar with electronic (762 of 1306 [58.3%]) vs paper (744 of 1305 [57.0%]) surveys. Practices enrolled 3 to 74 patients to the study, with survey completion rates ranging between 0% to 100%.

Respondents had a mean (SD) age of 61.0 (12.0) years at enrollment. Nearly half were female (210 [46.6%]), 33 (7.3%) identified as Black, 380 (84.3%) as White, and 38 (8.4%) as other race. A total of 14 (3.1%) were Hispanic or Latino. Nearly half had private insurance (224 [49.7%]). Patients were geographically distributed across the United States, with 189 (41.9%) in the Midwest, 26 (5.8%) in the Northeast, 121 (26.8%) in South, and 115 (25.5%) in the West. The enrollment median self-efficacy and financial worry COST scores were 7.2 (5.2-9.0) and 24.0 (14.0-34.0), respectively (Table 1).

**Table 1.** Baseline Characteristics in 451 Study Participants and Those Lost to Follow-Up at 24 Months

Characteristic^a^	Participants, No. (%)			*P* value^b^
	Baseline questions completed (N = 451)	24-mo Questions completed (n = 217)	24-mo Questions not completed (n = 234)	
Age, mean (SD), y	61.0 (12.0)	61.6 (12.0)	60.4 (12.0)	.28
Sex				
Male	241 (53.4)	120 (55.3)	121 (51.7)	.45
Female	210 (46.6)	97 (44.7)	113 (48.3)	
Race				
Black	33 (7.3)	10 (4.6)	23 (9.8)	.04
Other^c^	38 (8.4)	15 (6.9)	23 (9.8)	
White	380 (84.3)	192 (88.5)	188 (80.3)	
Ethnicity				
Hispanic or Latino	14 (3.1)	4 (1.8)	10 (4.3)	.14
Not Hispanic or Latino	426 (94.5)	208 (95.9)	218 (93.2)	
Not reported^d^	4 (0.9)	2 (0.9)	2 (0.9)	
Unknown^d^	7 (1.6)	3 (1.4)	4 (1.7)	
Education				
High school or less	178 (39.5)	64 (29.5)	114 (48.7)	<.001
College and advanced degree	268 (59.4)	152 (70.0)	116 (49.6)	
Not answered^d^	5 (1.1)	1 (0.5)	4 (1.7)	
Marital status				
Married, living with partner	297 (65.9)	146 (67.3)	151 (64.5)	.53
Unpartnered	152 (33.7)	70 (32.3)	82 (35.0)	
Not answered^d^	2 (0.4)	1 (0.5)	1 (0.4)	
Annual household income, $				
≤29 999	111 (24.6)	44 (20.3)	67 (28.6)	.03
30 000-59 999	135 (29.9)	62 (28.6)	73 (31.2)	
≥60 000	194 (43.0)	107 (49.3)	87 (37.2)	
Not answered^d^	11 (2.4)	4 (1.8)	7 (3.0)	
Employment				
Employed	215 (47.7)	104 (47.9)	111 (47.4)	.29
Retired	152 (33.7)	79 (36.4)	73 (31.2)	
Unemployed	78 (17.3)	32 (14.7)	46 (19.7)	
Not answered^d^	6 (1.3)	2 (0.9)	4 (1.7)	
Primary health insurance provider				
Private insurance	224 (49.7)	105 (48.4)	119 (50.9)	.07
Military, Indian Health Service, or Medicare	197 (43.7)	103 (47.5)	94 (40.2)	
Medicaid, single service, or no insurance	30 (6.7)	9 (4.1)	21 (9.0)	
Cancer type				
Colon cancer	290 (64.3)	138 (63.6)	152 (65.0)	.95
Rectal cancer	140 (31.0)	69 (31.8)	71 (30.3)	
Rectosigmoid junction	21 (4.7)	10 (4.6)	11 (4.7)	
Cancer stage				
I	69 (15.3)	42 (19.4)	27 (11.5)	.03
II	141 (31.3)	71 (32.7)	70 (29.9)	
III	241 (53.4)	104 (47.9)	137 (58.5)	
Receipt of chemotherapy				
No	184 (40.8)	102 (47.0)	82 (35.0)	.01
Yes	267 (59.2)	115 (53.0)	152 (65.0)	
Comorbidities, No.				
>1	240 (53.2)	112 (51.6)	128 (54.7)	.54
1	111 (24.6)	52 (24.0)	59 (25.2)	
0	100 (22.2)	53 (24.4)	47 (20.1)	
Region				
Midwest	189 (41.9)	92 (42.4)	97 (41.5)	<.001
Northeast	26 (5.8)	7 (3.2)	19 (8.1)	
South	121 (26.8)	45 (20.7)	76 (32.5)	
West	115 (25.5)	73 (33.6)	42 (17.9)	
ADI^e^				
Quartile 1, lowest deprivation (≤41.9)	119 (26.4)	66 (30.4)	53 (22.6)	NA
Quartile 2 (>41.9-44.5)	98 (21.7)	47 (21.7)	51 (21.8)	NA
Quartile 3 (>44.5-47.0)	122 (27.1)	58 (26.7)	64 (27.4)	NA
Quartile 4, highest deprivation (>47.0)	112 (24.8)	46 (21.2)	66 (28.2)	NA
Mean (SD)	44.1 (4.0)	43.7 (4.0)	44.6 (4.0)	.02
Median (IQR)	44.6 (41.5-47.0)	44.3 (40.5-46.7)	45.1 (42.2-47.2)	NA
Safety-net hospital				
Yes	66 (14.6)	27 (12.4)	39 (16.7)	.20
No or unknown	385 (85.4)	190 (87.6)	195 (83.3)	
Baseline self-efficacy, median (IQR)	7.2 (5.2-9.0)	7.8 (5.7-9.2)	6.5 (4.7-8.7)	<.001
Baseline COST median (IQR)^f^	24.0 (14.0-34.0)	28.0 (16.0-36.0)	21.0 (13.0-30.0)	<.001

Abbreviations: ADI, Area Deprivation Index; COST, Comprehensive Score for Financial Toxicity; NA, not applicable.

^a^ Demographic and other factors associated with cost-related care nonadherence and material hardship collected at baseline. Mean and SD were summarized for symmetric variables, median and IQR for asymmetric continuous variables, and frequencies and percentages for categorical variables.

^b^ *P* values compare participants with 24 months in the analysis set vs without 24 months. For continuous variables, the *P* value corresponds to the *t* test. For categorical variables, the *P* value corresponds to the χ^2^ test or the exact version of χ^2^ test, as appropriate.

^c^ American Indian or Alaska Native, Asian, multiracial, Native Hawaiian or Other Pacific Islander, not reported, and unknown are the subcategories included in the other category for race.

^d^ The *P* value for the comparison was performed after removing these categories.

^e^ The ADI is defined as neighborhood area deprivation index, with a higher score indicating greater neighborhood deprivation.

^f^ A total of 448 participants completed COST at baseline, with 215 completing it at 24 months and 233 not completing it at 24 months.

Comparing the characteristics of patients who did not complete the 24-month surveys (ie, were lost to follow-up) vs those who completed it, those lost to follow-up were more likely to be Black, have less education, and have lower income (Table 1). Additionally, they were more likely to have stage III cancer, receive chemotherapy, live in the Northeast or South, live in the most deprived neighborhoods, have lower self-efficacy scores, and have more financial worry (Table 1).

### Cost-Related Care Nonadherence and Its Associated Factors

Summary of care nonadherence and its contributing items at each time point are shown in Table 2. A total of 54 of 451 respondents (12.0%) at baseline and 20 of 217 respondents (9.2%) at 24 months reported care nonadherence. Of 217 patients with adherence data at baseline and 24 months, care nonadherence was 8.8% at baseline (19 participants) and 9.2% (20 participants) at 24 months (*P* = .84), with 12 patients (5.5%) showing improved adherence, 13 (6.0%) with worsening adherence, and 192 (88.5%) with no adherence change.

**Table 2.** Summary of Cost-Related Care Nonadherence and Material Hardship at Each Time Point

Outcome	Participants, No. (%)						
	Full sample					Patients who completed questionnaires at baseline and 24 mo (n = 217)	
	Baseline (N = 451)	3 mo (n = 268)	6 mo (n = 242)	12 mo (n = 208)	24 mo (n = 217)	Baseline	24 mo
Cost-related care nonadherence^a^							
No	397 (88.0)	237 (88.4)	217 (89.7)	186 (89.4)	197 (90.8)	198 (91.2)	197 (90.8)
Yes	54 (12.0)	31 (11.6)	25 (10.3)	22 (10.6)	20 (9.2)	19 (8.8)	20 (9.2)
Delay the filling of a prescription medication due to cost							
Missing	3 (0.7)	1 (0.4)	0	0	0	1 (0.5)	0
No	411 (91.1)	243 (90.7)	227 (93.8)	195 (93.8)	206 (94.9)	201 (92.6)	206 (94.9)
Yes	37 (8.2)	24 (9.0)	15 (6.2)	13 (6.3)	11 (5.1)	15 (6.9)	11 (5.1)
Filled only part of a prescription drug due to cost							
Missing	1 (0.2)	1 (0.4)	2 (0.8)	0	0	0	0
No	433 (96.0)	255 (95.1)	230 (95.0)	198 (95.2)	212 (97.7)	213 (98.2)	212 (97.7)
Yes	17 (3.8)	12 (4.5)	10 (4.1)	10 (4.8)	5 (2.3)	4 (1.8)	5 (2.3)
Stopped taking a medication due to cost							
Missing	2 (0.4)	1 (0.4)	0	1 (0.5)	1 (0.5)	1 (0.5)	1 (0.5)
No	438 (97.1)	259 (96.6)	234 (96.7)	201 (96.6)	206 (94.9)	212 (97.7)	206 (94.9)
Yes	11 (2.4)	8 (3.0)	8 (3.3)	6 (2.9)	10 (4.6)	4 (1.8)	10 (4.6)
Refused recommended tests due to cost							
Missing	2 (0.4)	0	0	1 (0.5)	0	1 (0.5)	0
No	435 (96.5)	261 (97.4)	233 (96.3)	197 (94.7)	209 (96.3)	213 (98.2)	209 (96.3)
Yes	14 (3.1)	7 (2.6)	9 (3.7)	10 (4.8)	8 (3.7)	3 (1.4)	8 (3.7)
Material condition^b^							
No	178 (39.5)	105 (39.2)	106 (43.8)	111 (53.4)	141 (65.0)	92 (42.4)	141 (65.0)
Yes	273 (60.5)	163 (60.8)	136 (56.2)	97 (46.6)	76 (35.0)	125 (57.6)	76 (35.0)
Decreased your basic spending on things like food and clothing due to cost							
Missing	1 (0.2)	0	2 (0.8)	0	2 (0.9)	0	2 (0.9)
No	275 (61.0)	154 (57.5)	151 (62.4)	151 (72.6)	176 (81.1)	142 (65.4)	176 (81.1)
Yes	175 (38.8)	114 (42.5)	89 (36.8)	57 (27.4)	39 (18.0)	75 (34.6)	39 (18.0)
Decreased your spending on leisure activities such as vacations, eating out, or movies due to the cost							
Missing	3 (0.7)	2 (0.7)	1 (0.4)	0	0	0	0
No	211 (46.8)	131 (48.9)	119 (49.2)	130 (62.5)	152 (70.0)	113 (52.1)	152 (70.0)
Yes	237 (52.5)	135 (50.4)	122 (50.4)	78 (37.5)	65 (30.0)	104 (47.9)	65 (30.0)
Used some or all of a savings account to pay for cancer care							
Missing	6 (1.3)	0	2 (0.8)	1 (0.5)	0	2 (0.9)	0
No	321 (71.2)	161 (60.1)	163 (67.4)	153 (73.6)	172 (79.3)	151 (69.6)	172 (79.3)
Yes	124 (27.5)	107 (39.9)	77 (31.8)	54 (26.0)	45 (20.7)	64 (29.5)	45 (20.7)
Patient or family member worked more to pay for cancer care							
Missing	6 (1.3)	1 (0.4)	1 (0.4)	2 (1.0)	1 (0.5)	1 (0.5)	1 (0.5)
No	412 (91.4)	225 (84.0)	215 (88.8)	183 (88.0)	199 (91.7)	198 (91.2)	199 (91.7)
Yes	33 (7.3)	42 (15.7)	26 (10.7)	23 (11.1)	17 (7.8)	18 (8.3)	17 (7.8)
Borrowed money to pay for cancer care							
Missing	3 (0.7)	2 (0.7)	1 (0.4)	0	0	1 (0.5)	0
No	413 (91.6)	239 (89.2)	220 (90.9)	197 (94.7)	204 (94.0)	203 (93.5)	204 (94.0)
Yes	35 (7.8)	27 (10.1)	21 (8.7)	11 (5.3)	13 (6.0)	13 (6.0)	13 (6.0)
Major material conditions^c^							
No	305 (67.6)	148 (55.2)	150 (62.0)	143 (68.8)	166 (76.5)	143 (65.9)	166 (76.5)
Yes	146 (32.4)	120 (44.8)	92 (38.0)	65 (31.3)	51 (23.5)	74 (34.1)	51 (23.5)
No. of major material conditions							
0	305 (67.6)	148 (55.2)	150 (62.0)	143 (68.8)	166 (76.5)	143 (65.9)	166 (76.5)
1	109 (24.2)	74 (27.6)	64 (26.4)	45 (21.6)	32 (14.7)	56 (25.8)	32 (14.7)
2	28 (6.2)	36 (13.4)	24 (9.9)	17 (8.2)	14 (6.5)	15 (6.9)	14 (6.5)
3	9 (2.0)	10 (3.7)	4 (1.7)	3 (1.4)	5 (2.3)	3 (1.4)	5 (2.3)
Minor material conditions^d^							
No	202 (44.8)	125 (46.6)	117 (48.3)	125 (60.1)	151 (69.6)	107 (49.3)	151 (69.6)
Yes	249 (55.2)	143 (53.4)	125 (51.7)	83 (39.9)	66 (30.4)	110 (50.7)	66 (30.4)
No. of minor material conditions							
0	202 (44.8)	125 (46.6)	117 (48.3)	125 (60.1)	151 (69.6)	107 (49.3)	151 (69.6)
1	86 (19.1)	37 (13.8)	39 (16.1)	31 (14.9)	28 (12.9)	41 (18.9)	28 (12.9)
2	163 (36.1)	106 (39.6)	86 (35.5)	52 (25.0)	38 (17.5)	69 (31.8)	38 (17.5)

^a^ Participants who answered yes to any of the cost-related care nonadherence questions, summarized by time point.

^b^ Participants who answered yes to any of the material condition questions, summarized by time point.

^c^ Major material conditions include using savings, borrowing money, and working more to pay for cancer care.

^d^ Minor material conditions include decreasing basic spending on food, clothing, and leisure activities to pay for cancer care.

Results of the univariable longitudinal model of nonadherence are in eTable 1 in Supplement 1. In a multivariable longitudinal model with time interaction, patients who were older (odds ratio [OR], 0.94; 95% CI: 0.91-0.98; *P* = .003) and had higher COST score (ie, less financially worried; OR, 0.90; 95% CI: 0.87-0.93; *P* < .001) were less likely to have nonadherence. Additionally, compared with male patients, female patients were less likely to have nonadherence over time (OR, 0.90; 95% CI: 0.85-0.96; *P* = .002) (Table 3; eFigure 1 in Supplement 1). Lastly, while at baseline patients with college or advanced education had lower nonadherence compared with those with high school education (OR, 0.34; 95% CI, 0.15-0.77; *P* = .009), over time, they become significantly more nonadherent than those with a high school degree (OR, 1.09; 95% CI, 1.03-1.17; *P* = .02) (Table 3; eFigure 2 in Supplement 1).

**Table 3.** Multivariable Longitudinal Model Result for Cost-Related Care Nonadherence With Time Interaction

Covariate	Odds ratio (95% CI)	*P* value	
		Estimate	Type III
Time, mo	0.99 (0.94-1.04)	.62	.77
Baseline COST	0.90 (0.87-0.93)	<.001	<.001
Age, y	0.94 (0.91-0.98)	.003	.003
Sex			
Female	1.32 (0.62-2.79)	.47	.47
Male			
Time × sex^a^			
Female	0.90 (0.85-0.96)	.002	.002
Male	1 [Reference]	NA	NA
Race			
Black	0.61 (0.19-2.00)	.42	.71
Other^b^	0.89 (0.23-3.46)	.86	
White	1 [Reference]	NA	NA
Education			
College and advanced degree	0.34 (0.15-0.77)	.009	.02
Not answered	3.94 (0.16-95.66)	.40	
High school or less	1 [Reference]	NA	NA
Time × education^a^			
College and advanced degree	1.09 (1.03-1.17)	.005	.02
Not answered	1.02 (0.56-1.84)	.96	
High school or less	1 [Reference]	NA	NA
Marital status			
Married, living with partner	0.96 (0.45-2.04)	.91	.91
Unpartnered or not answered	1 [Reference]	NA	NA
Region			
Midwest	1.63 (0.61-4.36)	.33	.67
Northeast	0.79 (0.15-4.08)	.78	
South	1.50 (0.46-4.93)	.51	
West	1 [Reference]	NA	NA
Annual household income, $			
30 000-59 999	0.71 (0.29-1.74)	.45	.47
≥60 000	0.44 (0.16-1.24)	.12	
Not answered	0.98 (0.10-9.57)	.99	
≤29 999	1 [Reference]	NA	NA
Primary health insurance provider			
Medicaid, single service, or no insurance	3.43 (1.04-11.31)	.04	.11
Military, Indian Health Service, or Medicare	1.83 (0.68-4.88)	.23	
Private insurance	1 [Reference]	NA	NA
Employment			
Employed	0.65 (0.28-1.50)	.31	.71
Retired	0.72 (0.23-2.22)	.57	
Not answered	1.63 (0.11-23.19)	.72	
Unemployed	1 [Reference]	NA	NA
Cancer type			
Rectal cancer	1.07 (0.52-2.20)	.84	.27
Rectosigmoid junction	3.17 (0.77-13.02)	.11	
Colon cancer	1 [Reference]	NA	NA
Cancer stage			
II	0.82 (0.28-2.44)	.73	.44
III	0.52 (0.16-1.69)	.28	
I	1 [Reference]	NA	NA
Receipt of chemotherapy			
Yes	0.95 (0.40-2.22)	.90	.90
No	1 [Reference]	NA	NA
Comorbidities, No.			
1	2.65 (0.93-7.55)	.07	.12
>1	2.59 (1.00-6.72)	.05	
None	1 [Reference]	NA	NA
ADI^c^	1.01 (0.91-1.13)	.83	.83

Abbreviations: ADI, Area Deprivation Index; COST, Comprehensive Score for Financial Toxicity; NA, not applicable.

^a^ For the odds ratio of the covariate (eg, sex) at a specific time point (eg, 24-month follow-up), multiply the odds ratio of that covariate (eg, 1.32) by the odds ratio of the interaction (eg, 0.90) to the power of the value of the specific time point (eg, 24 at 24-month follow-up), ie, 1.32 × 0.90^24^ = 1.32 × 0.08 = 0.11. Please note that for the calculation of the confidence interval, the covariance matrix between the estimate of the covariate and the estimate of the corresponding interaction needs to be used to first impute the variance and then calculate the confidence interval.

^b^ American Indian or Alaska Native, Asian, multiracial, Native Hawaiian or Other Pacific Islander, not reported, and unknown are the subcategories included in the other category for race.

^c^ Higher score means greater neighborhood deprivation.

### Material Hardship and Its Associated Factors

A total of 273 of 451 respondents (60.5%) at baseline and 76 of 217 respondents (35.0%) at 24 months reported material hardship (Table 2). Major hardship was reported by 146 (32.4%) and 51 (23.5%) at baseline and 24 months, respectively. Among 217 patients responding at baseline and 24 months, material hardship was reported by 125 (57.6%) at baseline and 76 (35.0%) at 24 months (*P* < .001), with 77 (35.5%) showing improved hardship, 35 (16.1%) showing worsening hardship, and 105 (48.4%) not reporting any change in their hardship. Of note, major material hardship was experienced by 74 (34.1%) at baseline compared with 51 (23.5%) at 24 months (*P* = .004), with 30 (13.8%) experiencing worsening of major hardship over 24 months. Our descriptive analysis showed worsening of major material hardship between baseline and 3 months with subsequent improvement. Minor material hardship improved over time (Table 2).

Results of univariable analysis of factors associated with material hardship are in eTable 2 in Supplement 1. In multivariable analysis, patients with higher COST score (ie, less financially worried; OR, 0.83; 95% CI, 0.80-0.86; *P* < .001) were less likely to have material hardship, and those who received chemotherapy were more likely to have material hardship (OR, 2.68; 95% CI, 1.15-6.29; *P* = .02). Over time, those employed were less likely to have material hardship (OR, 0.85; 95% CI, 0.78-0.93; *P* = .002), and those from safety-net hospitals (OR, 1.09; 95% CI, 1.01-1.17; *P* = .02) were more likely to have material hardship (Table 4; eFigures 3 and 4 in Supplement 1).

**Table 4.** Multivariable Longitudinal Model Result for Material Hardship With Time Interaction

Covariate	Odds ratio (95% CI)	*P* value	
		Estimate	Type III error
Time, mo	1.01 (0.94-1.09)	.80	.80
Baseline COST	0.83 (0.80-0.86)	<.001	<.001
Age, y	0.98 (0.94-1.01)	.22	.22
Sex			
Female	0.69 (0.37-1.28)	.24	.24
Male	1 [Reference]	NA	NA
Race			
Black	0.59 (0.16-2.22)	.43	.67
Other^a^	0.75 (0.22-2.54)	.64	
White	1 [Reference]	NA	NA
Region			
Midwest	0.75 (0.33-1.74)	.51	.83
Northeast	1.05 (0.22-4.96)	.95	
South	0.64 (0.22-1.86)	.41	
West	1 [Reference]	NA	NA
Annual household income, $			
30 000-59 999	0.95 (0.39-2.33)	.92	.93
≥60 000	0.86 (0.34-2.21)	.76	
Not answered	1.66 (0.20-13.51)	.64	
≤29 999	1 [Reference]	NA	NA
Primary health insurance provider			
Medicaid, single service, or no insurance	0.73 (0.18-2.98)	.66	.82
Military, Indian Health Service, or Medicare	0.79 (0.32-1.91)	.60	
Private insurance	1 [Reference]	NA	NA
Employment			
Employed	1.94 (0.66-5.72)	.23	.10
Retired	0.59 (0.18-1.95)	.39	
Not answered	0.98 (0.04-23.43)	.99	
Unemployed	1 [Reference]	NA	NA
Time × employment^b^			
Employed	0.85 (0.78-0.93)	<.001	.002
Retired	0.91 (0.83-0.99)	.03	
Not answered	1.00 (0.74-1.36)	.98	
Unemployed	1 [Reference]	NA	NA
Cancer type			
Rectal cancer	1.13 (0.57-2.24)	.73	.21
Rectosigmoid junction	3.86 (0.86-17.37)	.08	
Colon cancer	1 [Reference]	NA	NA
Cancer stage			
II	0.77 (0.29-2.02)	.59	.68
III	0.61 (0.20-1.86)	.38	
I	1 [Reference]	NA	NA
Receipt of chemotherapy			
Yes	2.68 (1.15-6.29)	.02	.02
No	1 [Reference]	NA	NA
Comorbidities, No.			
1	2.43 (0.98-6.01)	.06	.12
>1	2.02 (0.92-4.43)	.08	
None	1 [Reference]	NA	NA
Safety-net hospital			
Yes	0.54 (0.20-1.46)	.23	.23
No or unknown	1 [Reference]	NA	NA
Time × safety-net hospital^b^			
Yes	1.09 (1.01-1.17)	.02	.02
No or unknown	1 [Reference]	NA	NA
ADI^c^	1.02 (0.93-1.12)	.69	.69

Abbreviations: ADI, Area Deprivation Index; COST, Comprehensive Score for Financial Toxicity; NA, not applicable.

^a^ American Indian or Alaska Native, Asian, multiracial, Native Hawaiian or Other Pacific Islander, not reported, and unknown are the subcategories included in the other category for race.

^b^ For the odds ratio of the covariate (eg, employment) at a specific time point (eg, 24-month follow-up), multiply the odds ratio of that covariate (eg, 1.94) by the odds ratio of the interaction (eg, 0.85) to the power of the value of the specific time point (eg, 24 at 24-month follow-up), ie, 1.94 × 0.85^24^ = 1.94 × 0.02 = 0.04. Please note that for the calculation of confidence interval, the covariance matrix between the estimate of the covariate and the estimate of the corresponding interaction needs to be used to first impute the variance and then calculate the confidence interval.

^c^ A higher score means greater neighborhood deprivation.

### Assessment of Missing Data via IPW

The sensitivity analysis using IPW indicated that while estimates of regression coefficients were altered, significant covariates remained largely unchanged, except for the association of comorbidity with both outcomes. Our findings suggest that presence of comorbidity was associated with higher care nonadherence and material hardship (eTables 3 and 4 in Supplement 1).

## Discussion

In this prospective longitudinal cohort study of 451 adult patients with newly diagnosed early-stage colorectal cancer being treated in community oncology practices across the country, we found material hardship was more common than cost-related care nonadherence and decreased over time (57.6% at baseline vs 35.0% at 24 months). However, overall care nonadherence did not change significantly within 24 months of diagnosis (8.8% vs 9.2%).

To our knowledge, this is the first longitudinal study assessing financial hardship in early-stage cancer and more specifically, early-stage colorectal cancer. Our results are significant, as they confirm the longitudinal changes in financial hardship for patients with early-stage cancer are different than those with metastatic cancer. In a prior longitudinal study of material hardship in metastatic colorectal cancer, major material hardship increased from 24.9% to 71.3% over 12 months.^7^ However, in the current study, overall material hardship decreased from 60.5% at baseline to 46.6% at 12 months and to 35.0% at 24 months. Although some of this decrease is because patients with higher financial worry and higher risk factors for financial hardship were more likely to be lost to follow-up at 24 months (Table 1), the trend persists even among those who completed both baseline and 24-month surveys. None of the prior studies have assessed changes in care nonadherence in early or metastatic stage cancer. Our results confirm that for early-stage cancer, care nonadherence remained the same at diagnosis and during the cancer survivorship period.

Our study found that financial worry was the only modifiable variable that was associated with care nonadherence and material hardship. This finding suggests interventions addressing financial worry and anxiety (eg, cognitive behavioral interventions) may mitigate other domains of financial hardship. Of note, financial worry itself fluctuates throughout the cancer survivorship period with most patients showing improved financial worry over time, as shown in prior studies of patients with breast cancer,^9,10^ gynecological malignant neplasms,^23^ lung cancer,^24^ and early-stage colorectal cancer.^11^

Our results have implications for clinical practice. First, patients who dropped out were at higher risk for financial hardship. Therefore, early intervention is recommended. Second, as material hardship improves over time in early-stage cancer, earlier interventions, such as referral to financial navigation services, will likely be more helpful. Third, given a significant proportion of patients still experience material hardship (35.0%) and care nonadherence (9.2%) 2 years after diagnosis, financial hardship screening and referral to interventions should be longitudinal to be effective. Lastly, while younger age, lower education, receipt of chemotherapy, and higher financial worry were associated with risk for financial hardship at baseline, over time other factors may play a role. For example, men were more likely to have higher care nonadherence over time compared with women (eFigure 1 in Supplement 1; Table 3), while their material hardship was not different over time (data not shown), which is suggestive of superimposed sex-related behavioral difference in care adherence. Furthermore, those who were not employed or were receiving care at safety-net hospitals were more likely to experience higher material hardship over time, which suggests the need for longitudinal screening especially for populations at higher risk. Surprisingly, while higher education overall was associated with lower care nonadherence compared with those with high school degree, the nonadherence in this population increased over time (eFigure 2 in Supplement 1).

One of the strengths of our study was recruitment from community oncology practices across the United States where patients may not be routinely screened for financial hardship or may not have access to resources such as financial navigators. Approximately 55% of patients with cancer receive care in community practices,^25^ and only 50% of NCORP practices offer any financial navigator services.^26^

### Limitations

Our study has several limitations. First, the proportion of patients lost to follow-up in the current study was 61.6% at 24 months, which could be improved through higher patient engagement with incentives, use of patient navigators, development of data collection frameworks, and availability of different modalities to complete the surveys. Additionally, COVID-19 might have impacted our follow-up response rate. Of 432 patients enrolled prior to March 2020, 113 (25.1%) missed their 4 follow-up surveys vs 7 of 19 patients (36.8%) enrolled after March 2020. Those who dropped out of the study at 24 months reported more financial risk at baseline. Therefore, it is likely that the degree of material hardship and care nonadherence was underestimated. Although it is possible the differential lost to follow-up also impacted the trajectory of financial hardship over time, our analysis using IPW did show similar results. Second, our measures of care nonadherence and material hardship were self-reported and subject to recall bias. However, such self-reported measures have been used previously in national surveys, such as MEPS.^13,14,15^ Third, our survey was restricted to patients who spoke English, limiting the generalizability of our findings. Additionally, the ADI is calculated based on patients’ 5-digit zip code and may not have the granularity of ADI obtained from 9-digit zip codes.

## Conclusions

In patients with early-stage colorectal cancer, material hardship was more common than cost-related cancer care nonadherence and decreased significantly over time, while overall cost-related care nonadherence did not change. Approximately one-tenth of patients experienced cost-related care nonadherence 24 months after diagnosis and another one-third experienced material hardship. Early and longitudinal screening and referral to interventions are recommended to mitigate financial hardship and its consequences.

## Notes

Supplement 1. **eTable 1. **Univariate Longitudinal Model Result for Cost-Related Care Nonadherence **eTable 2.** Univariate Longitudinal Model Result for Material Hardship **eTable 3.** Multivariable Longitudinal Model Result for Cost-Related Care Nonadherence With Time Interaction (Inverse Probability Weighting Analysis) **eTable 4.** Multivariable Longitudinal Model Result for Material Hardship With Time Interaction (Inverse Probability Weighting Analysis) **eFigure 1.** Percentage of Cost-Related Care Nonadherence Over Time by Sex **eFigure 2.** Percentage of Cost-Related Care Nonadherence Over Time by Education **eFigure 3.** Percentage of Material Hardship Over Time by Employment **eFigure 4.** Percentage of Material Hardship Over Time by Safety-Net Hospital

Supplement 2.
Data Sharing Statement
