# Supplementary material for: Financial Hardship Among Patients With Early-Stage Colorectal Cancer
Source: JAMA Netw Open. 2024 Sep 17;7(9):e2431967. doi: 10.1001/jamanetworkopen.2024.31967 (PMC11409151; doi:10.1001/jamanetworkopen.2024.31967)
Supplement: Supplement 1. — eTable 1. Univariate Longitudinal Model Result for Cost-Related Care Nonadherence eTable 2. Univariate Longitudinal Model Result for Material Hardship eTable 3. Multivariable Longitudinal Model Result for Cost-Related Care Nonadherence With Time Interaction (Inverse Probability Weighting Analysis) eTable 4. Multivariable Longitudinal Model Result for Material Hardship With Time Interaction (Inverse Probability Weighting Analysis) eFigure 1. Percentage of Cost-Related Care Nonadherence Over Time by Sex eFigure 2. Percentage of Cost-Related Care Nonadherence Over Time by Education eFigure 3. Percentage of Material Hardship Over Time by Employment eFigure 4. Percentage of Material Hardship Over Time by Safety-Net Hospital [file jamanetwopen-e2431967-s001.pdf]

Supplemental Online Content

Sadigh G, Duan F, An N, et al. Financial hardship among patients with early-stage colorectal cancer. *JAMA Netw Open.* 2024;7(9):e2431967.
doi:10.1001/jamanetworkopen.2024.31967

eTable 1. Univariate Longitudinal Model Result for Cost-Related Care Nonadherence

eTable 2. Univariate Longitudinal Model Result for Material Hardship

eTable 3. Multivariable Longitudinal Model Result for Cost-Related Care Nonadherence With Time Interaction (Inverse Probability Weighting Analysis)

eTable 4. Multivariable Longitudinal Model Result for Material Hardship With Time Interaction (Inverse Probability Weighting Analysis)

eFigure 1. Percentage of Cost-Related Care Nonadherence Over Time by Sex

eFigure 2. Percentage of Cost-Related Care Nonadherence Over Time by Education

eFigure 3. Percentage of Material Hardship Over Time by Employment

eFigure 4. Percentage of Material Hardship Over Time by Safety-Net Hospital

This supplemental material has been provided by the authors to give readers additional information about their work.

© 2024 Sadigh G et al. *JAMA Network Open.*

eTable 1. Univariate Longitudinal Model Result for Cost-Related Care Nonadherence

Covariate	Category or Units	Odds Ratio (95% Confidence Interval)	P-value (Estimate)	P-value (Type 3)
**Baseline COST**				
Baseline COST		0.88 (0.85-0.91)	<.001	<.001
**Age**				
Age	Years	0.94 (0.91-0.97)	<.001	<.001
**Gender**				
Gender	Female	0.72 (0.34-1.56)	0.41	0.41
	Male [b]			
**Race**				
Race	Black	3.48 (0.92-13.23)	0.07	0.15
	Other[c]	0.68 (0.16-2.96)	0.61	
	White [b]			
**Education**				
Education	College and Advanced Degree	0.31 (0.14-0.68)	0.003	0.003
	Not Answered	8.14 (0.36-184.27)	0.19	
	High School or Less [b]			
**Marital Status**				
Marital Status	Married, Living with partner	0.43 (0.20-0.95)	0.04	0.04
	Unpartnered/Not Answered [b]			
**Region**				
Region	Midwest	2.13 (0.78-5.80)	0.14	0.02
	Northeast	2.63 (0.43-16.18)	0.30	
	South	5.57 (1.90-16.31)	0.002	
	West [b]			
**Annual Household Income**				
Annual Household Income	$30,000 to $59,999	0.29 (0.11-0.73)	0.009	<.001
	$60,000 and greater	0.11 (0.04-0.28)	<.001	
	Not Answered	0.40 (0.04-4.01)	0.43	
	Up to $29,999 [b]			
**Primary Health Insurance Provider**				
Primary Health Insurance Provider	Medicaid, Single service, No insurance	8.00 (2.00-32.06)	0.003	0.001
	Military, Indian, Medicare	0.53 (0.24-1.17)	0.11	
	Private insurance [b]			
**Employment**				
Employment	Employed	0.17 (0.06-0.46)	<.001	<.001
	Not Answered	0.69 (0.03-13.86)	0.81	
	Retired	0.07 (0.02-0.22)	<.001	
	Unemployed [b]			

© 2024 Sadigh G et al. *JAMA Network Open.*

Covariate	Category or Units	Odds Ratio (95% Confidence Interval)	P-value (Estimate)	P-value (Type 3)
**Cancer Type**				
Cancer Type	Rectal Cancer	1.43 (0.63-3.27)	0.40	0.47
	Rectosigmoid Junction	2.42 (0.45-13.06)	0.31	
	Colon Cancer [b]			
**Cancer Stage**				
Cancer Stage	Stage ll	1.68 (0.50-5.63)	0.40	0.70
	Stage lll	1.45 (0.46-4.50)	0.52	
	Stage l [b]			
**Receipt of Chemotherapy**				
Receipt of Chemotherapy	Yes	1.62 (0.74-3.57)	0.23	0.23
	No [b]			
**Comorbidities**				
Comorbidities	1	3.90 (1.17-13.01)	0.03	0.04
	>1	3.73 (1.28-10.93)	0.02	
	None [b]			
**Safety Net Hospital**				
Safety Net Hospital	Yes	1.29 (0.44-3.82)	0.65	0.65
	No/Unknown [b]			
**ADI[a] (Higher score means greater neighborhood deprivation)**				
ADI		1.14 (1.03-1.27)	0.010	0.010

Note:
There are 448 participants with baseline Cost-related care nonadherence, Material Hardship measures and available covariates included in the longitudinal mixed model.
[a] The ADI is defined as neighborhood area deprivation index, higher score means greater neighborhood deprivation.
[b] Reference group
[c] American Indian or Alaska Native, Asian, Multiple selected, Native Hawaiian or Other Pacific Islander, Not Reported, and Unknown are the subcategories included in the "Other" category for Race.

eTable 2. Univariate Longitudinal Model Result for Material Hardship

Covariate	Category or Units	Odds Ratio (95% Confidence Interval)	P-value (Estimate)	P-value (Type 3)
**Baseline COST**				
Baseline COST		0.83 (0.80-0.85)	<.001	<.001
**Age**				
Age	Years	0.91 (0.88-0.94)	<.001	<.001
**Gender**				
Gender	Female	0.61 (0.28-1.33)	0.21	0.21
	Male [b]			
**Race**				
Race	Black	7.29 (1.51-35.30)	0.01	0.047
	Other[c]	1.13 (0.28-4.54)	0.86	
	White [b]			
**Ethnicity**				
Ethnicity	Hispanic or Latino	0.30 (0.03-2.76)	0.28	0.43
	Not Reported/Unknown	0.38 (0.03-4.53)	0.45	
	Not Hispanic or Latino [b]			
**Education**				
Education	College and Advanced Degree	0.51 (0.23-1.12)	0.09	0.21
	Not Answered	1.87 (0.04-87.09)	0.75	
	High School or Less [b]			
**Marital Status**				
Marital Status	Married, Living with partner	0.64 (0.28-1.43)	0.27	0.27
	Unpartnered/Not Answered [b]			
**Region**				
Region	Midwest	1.64 (0.65-4.14)	0.30	0.010
	Northeast	7.15 (1.15-44.36)	0.03	
	South	4.88 (1.71-13.96)	0.003	
	West [b]			
**Annual Household Income**				
Annual Household Income	$30,000 to $59,999	0.27 (0.10-0.78)	0.02	0.004
	$60,000 and greater	0.16 (0.06-0.43)	<.001	
	Not Answered	0.20 (0.02-2.57)	0.22	
	Up to $29,999 [b]			
**Primary Health Insurance Provider**				
Primary Health Insurance Provider	Medicaid, Single service, No insurance	2.24 (0.45-11.07)	0.32	<.001
	Military, Indian, Medicare	0.12 (0.05-0.26)	<.001	
	Private insurance [b]			

© 2024 Sadigh G et al. *JAMA Network Open.*

Covariate	Category or Units	Odds Ratio (95% Confidence Interval)	P-value (Estimate)	P-value (Type 3)
**Employment**				
Employment	Employed	0.17 (0.06-0.49)	0.001	<.001
	Not Answered	0.37 (0.01-10.96)	0.56	
	Retired	0.02 (0.00-0.05)	<.001	
	Unemployed [b]			
**Cancer Type**				
Cancer Type	Rectal Cancer	2.95 (1.30-6.71)	0.01	0.01
	Rectosigmoid Junction	6.08 (0.95-38.86)	0.06	
	Colon Cancer [b]			
**Cancer Stage**				
Cancer Stage	Stage ll	3.66 (1.14-11.77)	0.03	<.001
	Stage lll	8.31 (2.75-25.12)	<.001	
	Stage 1 [b]			
**Receipt of Chemotherapy**				
Receipt of Chemotherapy	Yes	8.20 (3.67-18.31)	<.001	<.001
	No [b]			
**Comorbidities**				
Comorbidities	1	2.84 (0.93-8.67)	0.07	0.18
	>1	1.97 (0.76-5.12)	0.16	
	None [b]			
**Safety Net Hospital**				
Safety Net Hospital	Yes	1.02 (0.34-3.04)	0.98	0.98
	No/Unknown [b]			
**ADI[a] (Higher score means greater neighborhood deprivation)**				
ADI		1.13 (1.03-1.24)	0.01	0.01

Note:

There are 448 participants with baseline Cost-related care nonadherence, Material Hardship measures and available covariates included in the longitudinal mixed model.

[a] The ADI is defined as neighborhood area deprivation index, higher score means greater neighborhood deprivation.

[b] Reference group

[c] American Indian or Alaska Native, Asian, Multiple selected, Native Hawaiian or Other Pacific Islander, Not Reported, and Unknown are the subcategories included in the "Other" category for Race.

© 2024 Sadigh G et al. *JAMA Network Open.*

eTable 3. Multivariable Longitudinal Model Result for Cost-Related Care Nonadherence With Time Interaction (Inverse Probability Weighting Analysis)

Covariate	Category or Units	Odds Ratio (95% Confidence Interval)	P-value (Estimate)	P-value (Type 3)
**Time**	Months	1.01 (0.96, 1.06)	0.62	0.89
**Baseline COST**		0.86 (0.81, 0.91)	<.001	<.001
**Age**	Years	0.92 (0.86, 0.98)	0.008	0.008
**Sex**	Female	1.53 (0.50, 4.66)	0.45	0.45
	Male [b]			
**Time*Sex**	Female	0.87 (0.82, 0.93)[d]	<.001	<.001
	Male [b]			
**Race**	Black	0.28 (0.04, 1.93)	0.20	0.43
	Other[c]	0.82 (0.10, 6.53)	0.85	
	White [b]			
**Education**	College and Advanced Degree	0.25 (0.08, 0.82)	0.02	0.04
	Not Answered	5.91 (0.04, 809.89)	0.48	
	High School or Less [b]			
**Time*Education**	College and Advanced Degree	1.11 (1.04, 1.18)	<.00	0.004
	Not Answered	1.01 (0.47, 2.16)	0.99	
	High School or Less [b]			
**Marital Status**	Married, Living with partner	1.03 (0.31, 3.37)	0.96	0.96
	Unpartnered/Not Answered [b]			
**Region**	Midwest	2.00 (0.43, 9.39)	0.38	0.55
	Northeast	0.42 (0.03, 5.78)	0.52	
	South	1.85 (0.28, 12.03)	0.52	
	West [b]			
**Annual Household Income**	$30,000 to $59,999	0.59 (0.14, 2.39)	0.46	0.37
	$60,000 and greater	0.26 (0.05, 1.32)	0.10	
	Not Answered	1.71 (0.05, 55.06)	0.76	
	Up to $29,999 [b]			
**Primary Health Insurance Provider**	Medicaid, Single service, No insurance	7.10 (1.07, 46.96)	0.04	0.12
	Military, Indian, Medicare	2.13 (0.46, 9.81)	0.33	
	Private insurance [b]			
**Employment**	Employed	0.44 (0.12, 1.64)	0.22	0.63
	Retired	0.54 (0.09, 3.13)	0.49	
	Not Answered	1.56 (0.03, 93.11)	0.83	
	Unemployed [b]			
**Cancer Type**	Rectal Cancer	1.08 (0.35, 3.36)	0.90	0.63
	Rectosigmoid Junction	3.10 (0.31, 31.11)	0.34	
	Colon Cancer [b]			
**Cancer Stage**	Stage ll	1.26 (0.22, 7.13)	0.79	0.51
	Stage lll	0.59 (0.09, 3.89)	0.58	
	Stage l [b]			

© 2024 Sadigh G et al. *JAMA Network Open.*

Covariate	Category or Units	Odds Ratio (95% Confidence Interval)	P-value (Estimate)	P-value (Type 3)
**Receipt of Chemotherapy**	Yes	0.68 (0.18, 2.60)	0.57	0.57
	No [b]			
**Comorbidities**	1	8.36 (1.57, 44.62)	0.01	0.04
	>1	5.38 (1.17, 24.65)	0.03	
	None [b]			
**ADI[s] (Higher score means greater neighborhood deprivation)**		1.01 (0.85, 1.21)	0.88	0.88

Note:
There are 448 participants with baseline Cost-related care non-adherence, Material Hardship measures and available covariates included in the longitudinal mixed model.
[a] The ADI is defined as neighborhood area deprivation index, higher score means greater neighborhood deprivation.
[b] Reference group
[c] American Indian or Alaska Native, Asian, Multiple selected, Native Hawaiian or Other Pacific Islander, Not Reported, and Unknown are the subcategories included in the "Other" category for Race.
[d] For the odds ratio of the covariate (e.g., sex) at a specific time point (e.g., 24-month FU), multiply the odds ratio of that covariate (e.g., 1.53) by the odds ratio of the interaction (e.g., 0.87) to the power of the value of the specific time point (e.g., 24 at 24-month FU), i.e., 1.53 * (0.87 ** 24) = 1.53 * 0.035 =0.054. Please note that for the calculation of confidence interval, the covariance matrix between the estimate of the covariate and the estimate of the corresponding interaction needs to be used to first impute the variance, and then calculate the confidence interval.

© 2024 Sadigh G et al. *JAMA Network Open.*

eTable 4. Multivariable Longitudinal Model Result for Material Hardship With Time Interaction (Inverse Probability Weighting Analysis)

Covariate	Category or Units	Odds Ratio (95% Confidence Interval)	P-value (Estimate)	P-value (Type 3)
Time	Months	1.03 (0.96, 1.11)	0.41	0.78
Baseline COST		0.77 (0.73, 0.82)	<.001	<.001
Age	Years	0.96 (0.91, 1.02)	0.18	0.18
Sex	Female	0.46 (0.19, 1.14)	0.09	0.09
	Male [b]			
Race	Black	0.46 (0.07, 2.96)	0.41	0.71
	Other[c]	0.88 (0.15, 5.03)	0.88	
	White [b]			
Region	Midwest	0.78 (0.23, 2.66)	0.69	0.88
	Northeast	1.24 (0.13, 11.36)	0.85	
	South	0.59 (0.12, 2.82)	0.51	
	West [b]			
Annual Household Income	$30,000 to $59,999	0.86 (0.24, 3.09)	0.82	0.95
	$60,000 and greater	0.85 (0.22, 3.31)	0.81	
	Not Answered	1.97 (0.09, 42.92)	0.67	
	Up to $29,999[b]			
Primary Health Insurance Provider	Medicaid, Single service, No insurance	0.66 (0.09, 4.77)	0.68	0.90
	Military, Indian, Medicare	0.84 (0.23, 3.05)	0.79	
	Private insurance [b]			
Employment	Employed	2.54 (0.58, 11.10)	0.22	0.13
	Retired	0.51 (0.10, 2.67)	0.42	
	Not Answered	1.20 (0.01, 106.66)	0.94	
	Unemployed [b]			
Time*Employment	Employed	0.80 (0.73, 0.86)[d]	<.001	<.001
	Retired	0.87 (0.80, 0.95)	0.001	
	Not Answered	0.95 (0.67, 1.33)	0.76	
	Unemployed [b]			
Cancer Type	Rectal Cancer	0.89 (0.33, 2.41)	0.82	0.30
	Rectosigmoid Junction	5.11 (0.59, 43.88)	0.14	
	Colon Cancer [b]			
Cancer Stage	Stage ll	0.70 (0.17, 2.88)	0.62	0.47
	Stage lll	0.39 (0.08, 1.96)	0.25	
	Stage 1 [b]			
Receipt of Chemotherapy	Yes	5.99 (1.75, 20.48)	0.004	0.004
	No [b]			
Comorbidities	1	3.97 (1.06, 14.94)	0.04	0.11
	>1	2.68 (0.86, 8.41)	0.09	
	None [b]			
Safety Net Hospital	Yes	0.42 (0.11, 1.64)	0.21	0.21
	No/Unknown [b]			

© 2024 Sadigh G et al. *JAMA Network Open.*

Covariate	Category or Units	Odds Ratio (95% Confidence Interval)	P-value (Estimate)	P-value (Type 3)
Time*Safety Net Hospital	Yes	1.13 (1.06, 1.21)	<.001	<.001
	No/Unknown [b]			
ADI[a] (Higher score means greater neighborhood deprivation)		1.02 (0.89, 1.17)	0.79	0.79

Note:
There are 448 participants with baseline Cost-related care non-adherence, Material Hardship measures and available covariates included in the longitudinal mixed model.
[a] The ADI is defined as neighborhood area deprivation index, higher score means greater neighborhood deprivation.
[b] Reference group
[c] American Indian or Alaska Native, Asian, Multiple selected, Native Hawaiian or Other Pacific Islander, Not Reported, and Unknown are the subcategories included in the "Other" category for Race.
[d] For the odds ratio of the covariate (e.g., Employment) at a specific time point (e.g., 24-month FU), multiply the odds ratio of that covariate (e.g., 2.54) by the odds ratio of the interaction (e.g., 0.80) to the power of the value of the specific time point (e.g., 24 at 24-month FU), i.e., 2.54 * (0.80 ** 24) = 2.54 *0.0047=0.012 Please note that for the calculation of confidence interval, the covariance matrix between the estimate of the covariate and the estimate of the corresponding interaction needs to be used to first impute the variance, and then calculate the confidence interval.

© 2024 Sadigh G et al. *JAMA Network Open.*

eFigure 1. Percentage of Cost-Related Care Nonadherence Over Time by Sex

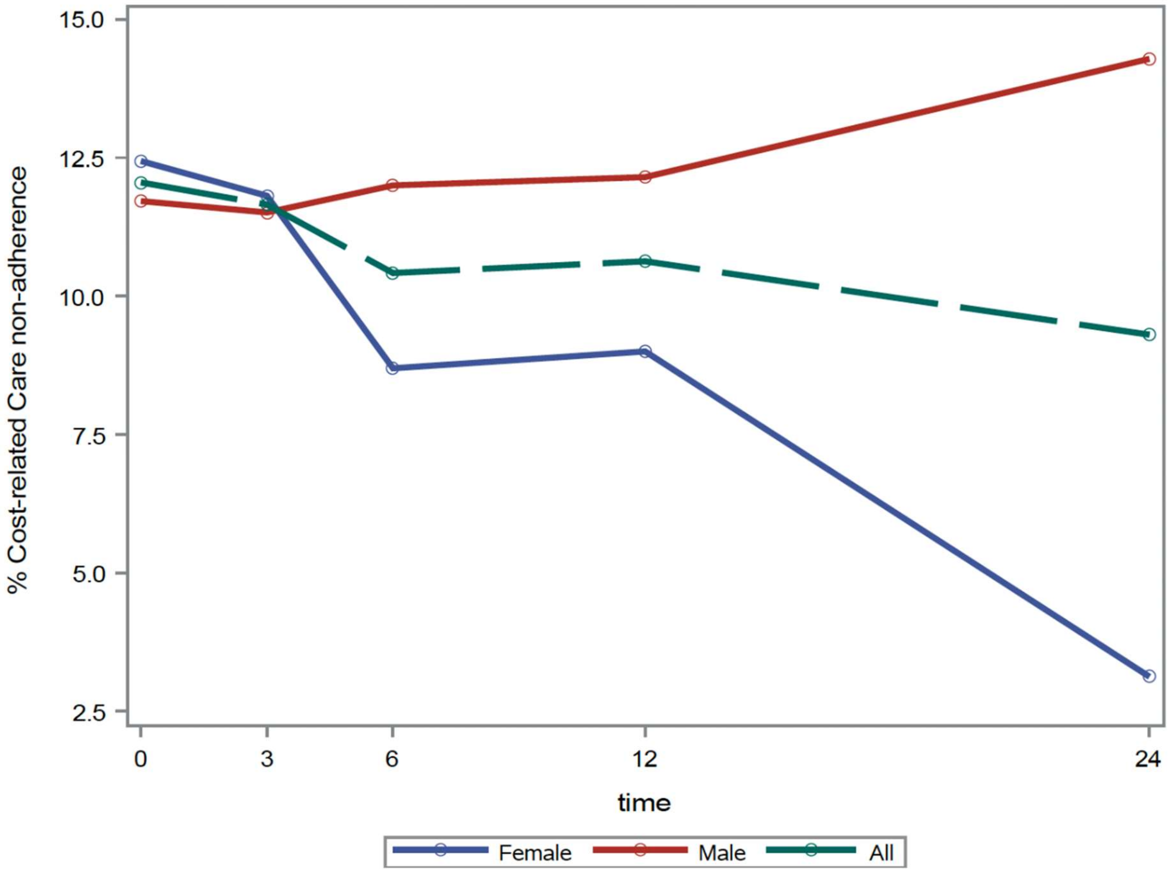

© 2024 Sadigh G et al. *JAMA Network Open.*

eFigure 2. Percentage of Cost-Related Care Nonadherence Over Time by Education

Note: Participants categorized as "Not Answered" for their education level have been excluded from this figure due to the limited frequency

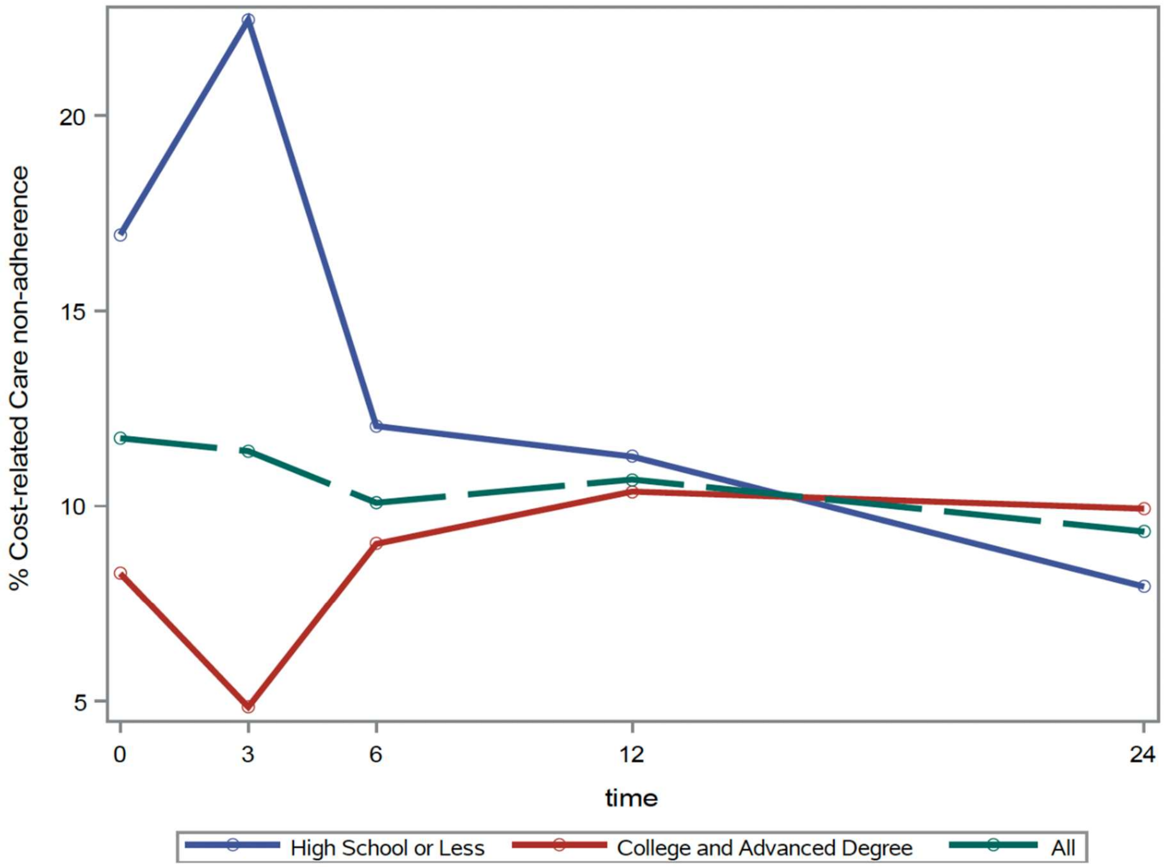

© 2024 Sadigh G et al. *JAMA Network Open.*

eFigure 3. Percentage of Material Hardship Over Time by Employment

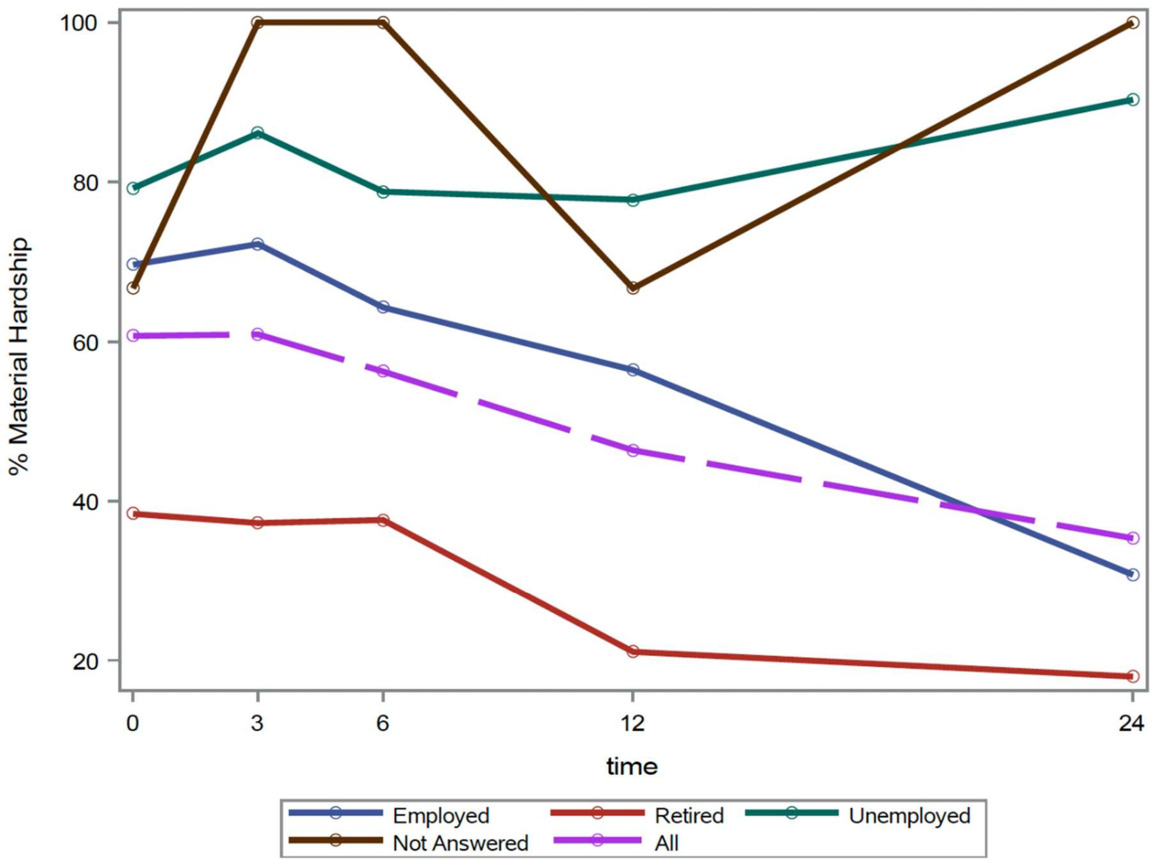

© 2024 Sadigh G et al. *JAMA Network Open.*

eFigure 4. Percentage of Material Hardship Over Time by Safety-Net Hospital

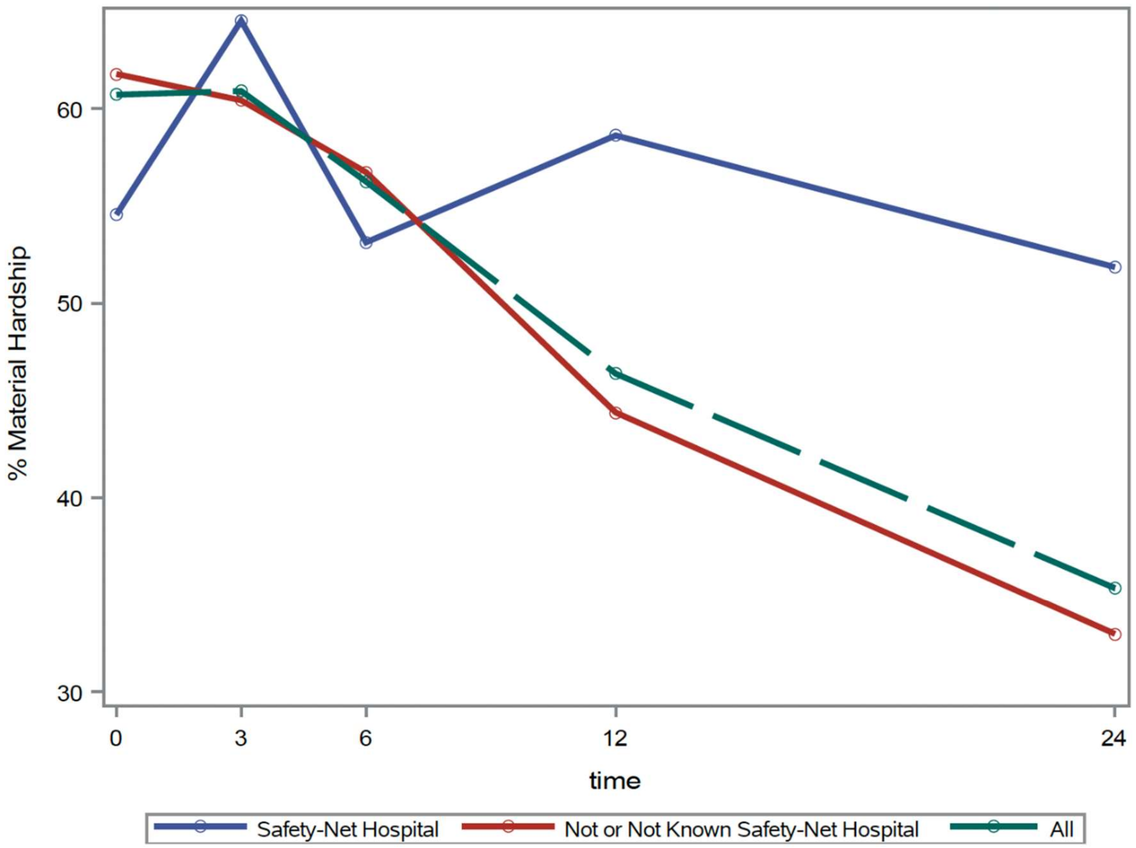

© 2024 Sadigh G et al. *JAMA Network Open.*
