# Supplementary material for: Financial Hardship Among Patients With Early-Stage Colorectal Cancer
Source: JAMA Netw Open. 2024 Sep 17;7(9):e2431967. doi: 10.1001/jamanetworkopen.2024.31967 (PMC11409151; doi:10.1001/jamanetworkopen.2024.31967)
Supplement: Supplement 2. — Data Sharing Statement [file jamanetwopen-e2431967-s002.pdf]

Data Sharing Statement

Sadigh. Financial Hardship Among Patients With Early-Stage Colorectal Cancer. *JAMA Netw Open*. Published September 09, 2024. doi:10.1001/jamanetworkopen.2024.31967

Data
Data available: Yes
Data types: Deidentified participant data
How to access data: Data request would be sent to ECOG ACRIN communications@ecog-acrin.org
When available: With publication

Supporting Documents
Document types: None

Additional Information
Who can access the data: Researchers whose proposed use of the data has been approved via ECOG ACRIN policy.
Types of analyses: For research related projects
Mechanisms of data availability: After approval of a proposal by ECOG ACRIN and signed EA data access agreement.
